# Fucoxanthin, a Marine Carotenoid Present in Brown Seaweeds and Diatoms: Metabolism and Bioactivities Relevant to Human Health

**DOI:** 10.3390/md9101806

**Published:** 2011-10-10

**Authors:** Juan Peng, Jian-Ping Yuan, Chou-Fei Wu, Jiang-Hai Wang

**Affiliations:** Guangdong Provincial Key Laboratory of Marine Resources and Coastal Engineering, School of Marine Sciences, Sun Yat-Sen University, Guangzhou 510275, China; E-Mails: pengj28@mail.sysu.edu.cn (J.P.); hyh-409@163.com (C.-F.W.)

**Keywords:** fucoxanthin, metabolite, bioactivity, brown seaweed, diatom

## Abstract

The marine carotenoid fucoxanthin can be found in marine brown seaweeds, the macroalgae, and diatoms, the microalgae, and has remarkable biological properties. Numerous studies have shown that fucoxanthin has considerable potential and promising applications in human health. In this article, we review the current available scientific literature regarding the metabolism, safety, and bioactivities of fucoxanthin, including its antioxidant, anti-inflammatory, anticancer, anti-obese, antidiabetic, antiangiogenic and antimalarial activities, and its protective effects on the liver, blood vessels of the brain, bones, skin, and eyes. Although some studies have shown the bioavailability of fucoxanthin in brown seaweeds to be low in humans, many studies have suggested that a dietary combination of fucoxanthin and edible oil or lipid could increase the absorption rate of fucoxanthin, and thus it might be a promising marine drug.

## 1. Introduction

**Figure 1:**
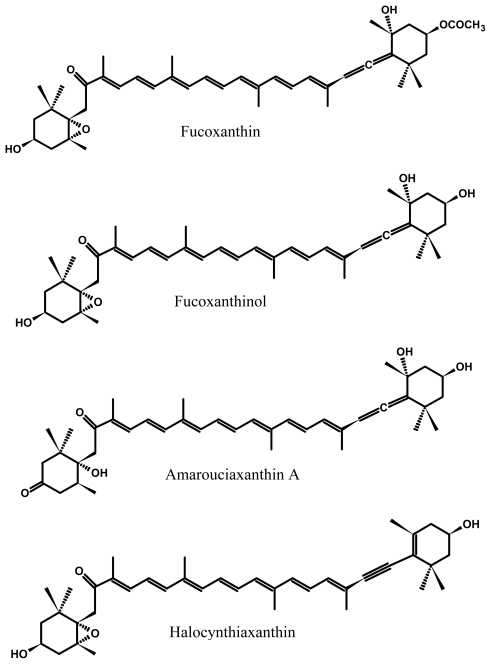
Structures of fucoxanthin and its metabolites fucoxanthinol, amarouciaxanthin A, and halocynthiaxanthin.

Fucoxanthin is one of the most abundant carotenoids, and contributes more than 10% of the estimated total production of carotenoids in nature, especially in the marine environment [1]. Fucoxanthin is an orange-colored pigment, along with chlorophylls *a* and *c* and β-carotene, present in *Chromophyta* (*Heterokontophyta* or *Ochrophyta*), including brown seaweeds (*Phaeophyceae*) and diatoms (*Bacillariophyta*) [2,3]. Fucoxanthin was first isolated from the marine brown seaweeds *Fucus*, *Dictyota*, and *Laminaria* by Willstätter and Page [4] in 1914. The complete structure (Figure 1) of fucoxanthin including chirality was determined by Englert *et al.* [5]. Some brown seaweeds are the most common edible macroalgae in South-East Asia and a few European countries [6]. Diatoms are unicellular planktonic microalgae and exhibit a characteristic golden-brown color due to a high amount of fucoxanthin that plays a major role in the light-harvesting complex of photosystems [7].

Fucoxanthin has been isolated for its bioactivity studies from the marine brown seaweeds *Alaria crassifolia* [8], *Cladosiphon okamuranus* [9], *Cystoseira hakodatensis* [8,10], *Eisenia bicyclis* [8], *Fucus serratus* [11], *Fucus vesiculosus* [12], *Hijikia fusiformis* [13,14], *Ishige okamurae* [15], *Kjellmaniella crassifolia* [8], *Laminaria japonica* [16–18], *Laminaria ochotensis* [18], *Myagropsis myagroides* [19], *Padina tetrastromatica* [6], *Petalonia binghamiae* [20], *Sargassum fulvellum* [14], *Sargassum heterophyllum* [21], *Sargassum horneri* [10], *Sargassum siliquastrum* [22], and *Undaria pinnatifida* [10,14,23–32], and the diatoms *Chaetoseros* sp. [33,34], *Cylindrotheca closterium* [35], *Odontella aurita* [36], and *Phaeodactylum tricornutum* [2].

Fucoxanthin has remarkable biological properties based on its unique molecular structure similar to neoxanthin, dinoxanthin, and peridinin, which is different from that of other carotenoids such as β-carotene and astaxanthin. Fucoxanthin has an unusual allenic bond and some oxygenic functional groups such as epoxy, hydroxyl, carbonyl and carboxyl moieties in its molecule (Figure 1) that contribute to its unique structure [2,14]. The allenic bond was found mainly in carotenoids such as fucoxanthin, which was the first allenic carotenoid found in brown seaweeds [1], and was responsible for the higher antioxidant [27].

This article reviews the current available scientific literature regarding the metabolism, safety, and bioactivities of fucoxanthin, including its antioxidant, anti-inflammatory, anticancer, anti-obese, antidiabetic, antiangiogenic and antimalarial activities, and its protective effects on the liver, blood vessels of the brain, bones, skin, and eyes.

## 2. Bioavailability, Metabolism, and Safety of Fucoxanthin

### 2.1. Bioavailability and Metabolism of Fucoxanthin

Solubilization of carotenoids in mixed micelles is considered to be requirement for absorption by intestinal cells. Sugawara *et al*. [37] demonstrated that phospholipids had intense effects on carotenoid absorption, and the cellular absorption was dependent on the lipophilicity of carotenoids. This study suggested that pancreatic phospholipase A_2_ and lysophosphatidylcholine were crucial in modulating the absorption of carotenoids in the gastrointestinal tract and supported a simple diffusion mechanism for carotenoid assimilation by the intestinal epithelium. It was suggested that fucoxanthin was more readily absorbed than lutein esters in mouse intestine [38] and its metabolites were accumulated in the body at a higher ratio than astaxanthin [39].

It had been previously reported that ingested fucoxanthin was totally deacetylated in the instestinal lumen and transported via blood in White leghorn laying hens fed the brown seaweed *F. serratus*, and thus fucoxanthinol was present as one of the main carotenoids in egg yolk [11]. Sugawara *et al*. [40] showed that fucoxanthin was hydrolyzed to fucoxanthinol during the uptake by differentiated Caco-2 cells, a tissue culture model for studying the absorption of dietary compounds by human intestinal epithelium. This study also showed fucoxanthinol appeared in mouse plasma after ingestion of fucoxanthin, indicating that dietary fucoxanthin was deacetylated into fucoxanthinol in the intestinal tract by lipase and esterase from the pancreas or in intestinal cells, and incorporated as fucoxanthinol from the digestive tract into the blood circulation system in mammals. Therefore, the bioavailability of fucoxanthinol is higher than that of fucoxanthin in the body [41].

Asai *et al*. [42] investigated further biotransformation of fucoxanthinol in ICR mice and HepG2 cells, and revealed that an unknown metabolite had been previously found in marine tunicate *Amaroucium pliciferum*, and thus was identified as amarouciaxanthin A (Figure 1). The bioconversion of fucoxanthinol into amarouciaxanthin A through dehydrogenation/isomerization was principally shown in liver microsomes, and fucoxanthinol supplemented to culture medium via HepG2 cells was also converted into amarouciaxanthin A. Therefore, it was suggested that fucoxanthin was converted to fucoxanthinol in the gastrointestinal tract and was then metabolized to amarouciaxanthin A in the liver [6,10,39,42–44], and no fucoxanthin was detected in plasma and liver [42,44]. Hashimoto *et al*. [39] found that fucoxanthinol and amarouciaxanthin A were detectable in plasma, erythrocytes, the liver, lungs, kidney, heart, spleen, and adipose tissue in ICR mice fed 160 nmol fucoxanthin, whereas no fucoxanthin was detected in these tissues or in the blood.

However, the daily oral administration of fucoxanthin for 1 week showed that a small amount of fucoxanthin was not metabolized and was detectable in the liver, lung, kidney, heart, spleen, and adipose tissue of the mice [39]. The percentage of fucoxanthin, fucoxanthinol, and amarouciaxanthin A to the sum of all of these in the adipose tissue was 13, 32, and 55%, respectively, while the percentage in the other tissues was 1–11, 63–76 and 20–26%, respectively [39], indicating that amarouciaxanthin A accumulated preferentially in the adipose tissue [10,38,39], while fucoxanthinol accumulated mainly in the liver, lungs, kidney, heart, and spleen [39]. Yonekura *et al*. [38] showed that fucoxanthinol and amarouciaxanthin A accumulated mainly in adipose tissues (3.13–3.64 μmol/kg), in which the contents were 2.2- to 2.6-fold of that in plasma, liver, and kidney (1.29–1.80 μmol/kg) in ICR mice fed 128 nmol fucoxanthin daily for 14 days.

Further transformation of amarouciaxanthin A to more polar metabolites might be taking place in ICR mice [38]. Sangeetha *et al*. [6] reported in detail on various metabolites of fucoxanthin besides the major metabolites fucoxanthinol and amarouciaxanthin A in rats, proposed a possible metabolic pathway of fucoxanthin in the plasma and liver of rats, and speculated that these metabolites might be formed as a result of enzymatic reactions such as isomerization, dehydrogenation, deacetylation, oxidation, and demethylation.

In addition, in marine animals such as oysters and clams [45–47], fucoxanthinol was further metabolized into halocynthiaxanthin (Figure 1), which was first isolated from sea squirt *Halocynthia roretzi* [48,49].

In a human study, Asai *et al*. [28] determined the plasma concentrations of fucoxanthin metabolites before and after 1-week dietary interventions with *U. pinnatifida* to estimate the intestinal absorption of fucoxanthin in five healthy volunteers. The results showed that the plasma concentration of fucoxanthinol was low (0.8 nmol/L) after 1 week of uptake (6.1 mg fucoxanthin/day), and no fucoxanthin and amarouciaxanthin A was detected in the plasma, and it was possible that some components (e.g., dietary fibre) in algal matrix inhibited the intestinal absorption of fucoxanthin.

It has been suggested that dietary combination of fucoxanthin and edible oil or lipid could increase the absorption rate of fucoxanthin in KK-Ay mice [32,50,51] and in obese premenopausal women [52].

### 2.2. Safety of Fucoxanthin

Fucoxanthin and fucoxanthinol had few adverse effects on normal and uninfected cells both *in vitro* and *in vivo* [53,54]. Zaragozá *et al*. [12] studied the toxicity of extracts from *F. vesiculosus* in mice and rats, and indicated that even at the dose of 750 mg/kg daily for 4 weeks, no any relevant signs of toxicity occurred. Kadekaru *et al*. [55] conducted a toxicity study on the repeated oral dosing of fucoxanthin (95% purity) to rats for 28 days, and revealed that fucoxanthin did not show obvious toxicity.

Beppu *et al*. [3] conducted a single dose toxicity study at doses of 1000 and 2000 mg/kg and a repeated oral dose toxicity study at doses of 500 and 1000 mg/kg for 30 days on purified fucoxanthin (93% purity) in ICR mice. The results showed that no mortality and no abnormalities in gross appearance were found in both studies, and no abnormal changes in liver, kidney, spleen, and gonadal tissues induced by fucoxanthin in the histological observations of the repeated doses study. Subsequently, Beppu *et al*. [56] indicated that fucoxanthin had no genotoxic/mutagenic effect on the bone marrow cells of mice. Iio *et al*. [33,34] investigated the subchronic toxicity of fucoxanthin in rats and genotoxicity in mice. In the single oral dose study, no mortality and no change were observed. The 50% lethal dose of fucoxanthin was more than 2000 mg/kg body weight. In the 13-week oral dose study, the no-observed-adverse-effect level of fucoxanthin was 200 mg/kg body weight under the tested subchronic dose condition. These studies suggested that fucoxanthin was a safe compound and did not exhibit toxicity and mutagenicity under these experimental conditions [3,34,56].

However, some carotenoids may have ability to increase circulating cholesterol level in rodents as a common feature [3]. Although fucoxanthin markedly elevated plasma high-density lipoprotein cholesterol level [3,55,57], total cholesterol level in the blood significantly increased to the same degree while the dose of fucoxanthin was 50 mg/kg daily for 28 days in Crl:CD (SD) rat and 1000 mg/kg daily for 30 days in ICR mice [3,55]. To further ascertain the safety of fucoxanthin, the mechanism by which fucoxanthin induces hypercholesterolemia and species differences should be elucidated.

## 3. Potential Health-Promoting Effects of Fucoxanthin

### 3.1. Antioxidant Activity

Antioxidant activity is one of the important characteristics of carotenoids, and many of their biological effects are related to the ability to scavenge reactive oxygen species, which is one of the factors for their disease preventing effects [27]. Earlier studies had indicated that fucoxanthin was also an effective radical scavenger [2,14]. The chemically generated radicals used to evaluate the antioxidant activity of fucoxanthin include DPPH (1,1-diphenyl-2-picrylhydrazyl), 12-DS (12-doxyl-stearic acid), NB-L (the radical adduct of nitrosobenzene with linolenic acid radical), AAPH (2,2′-azo-bis- (2-amidinopropane) dihydrochloride), ABTS (2,2′-azinobis-3-ethylbenzothiazoline-6-sulphonate), and ABAP (2,2′-azo-bis-2-amidinopropane) [8,12,13,27].

It has been reported that the extracts of *H. fusiformis*, *C. okamuranus*, *U. pinnatifida*, and *S. fulvellum* showed a strong DPPH radical scavenging activity [9,14]. Recently, Airanthi *et al*. [8] showed that the methanol extract of *C. hakodatensis* was a good source for antioxidants and the high antioxidant activity was based not only on the high content of phenolics, but also on the presence of fucoxanthin, demonstrating the synergy in the antioxidant activity of phenolics and fucoxanthin. Although two extracts from *F. vesiculosus* containing 28.8% polyphenols or 18% polyphenols and 0.0012% fucoxanthin, respectively, exhibited antioxidant activity in noncellular systems (O_2_ ^•−^, DPPH, ABTS, and ABAP) and in activated RAW 264.7 macrophages, as well as in ex vivo assays in plasma and erythrocytes, after the 4-week treatment in rats, only extract containing fucoxanthin showed an antioxidant activity *ex vivo* through preventing oxidant formation, scavenging superoxide anion (O_2_ ^•−^), and reducing active intermediates [12].

It was shown that fucoxanthin scavenged organic free radicals 12-DS, DPPH, and NB-L with the inhibitions of 66% (incubation for 12 h), 28% (1 h), and 57% (12 h), respectively, in the electron spin resonance signal intensities [13]. Fucoxanthin could effectively inhibit intracellular reactive oxygen species formation, DNA damage, and apoptosis induced by H_2_O_2_, possibly due to the ability of fucoxanthin to increase catalase [22].

Sachindra *et al*. [27] assessed the antioxidant activities of fucoxanthin and its two metabolites, fucoxanthinol and halocynthiaxanthin, in vitro with respect to radical scavenging (DPPH, ABTS, hydroxyl radical, and superoxide radical) and singlet oxygen quenching abilities, and suggested that fucoxanthin and fucoxanthinol exhibited antioxidant activities higher or similar to that of α-tocopherol, and halocynthiaxanthin showed comparatively lower antioxidant activities. The hydroxyl radical scavenging activity of fucoxanthin was 7.9, 16.3, and 13.5 times higher than that by fucoxanthinol, halocynthiaxanthin, and α-tocopherol, respectively, but singlet oxygen quenching ability of fucoxanthin was lower than that of β-carotene. Although halocynthiaxanthin showed very poor scavenging activity, of 19 carotenoids including halocynthiaxanthin, fucoxanthinol, astaxanthin, canthaxanthin, lutein, and β-carotene, halocynthiaxanthin showed the highest suppressive effect on superoxide anion (O_2_ ^•−^) and nitric oxide (NO) generation from differentiated human promyelocytic HL-60 cells and mouse macrophage RAW 264.7 cells, respectively [45].

Burton and Ingold [58] previously pointed out that β-carotene exhibited good radical-trapping antioxidant activity only at partial pressures of oxygen significantly less than the pressure of oxygen in normal air, which is found in most tissues under physiological conditions. Nomura *et al*. [2] found that, under anoxic conditions, fucoxanthin equimolarly reacted with DPPH as radical quencher, whereas β-carotene, β-cryptoxanthin, zeaxanthin, licopene, and lutein scarcely reacted with DPPH and had practically no quenching activities, suggesting that fucoxanthin was more reactive to radicals than other carotenoids under anoxic conditions. However, under aerobic conditions, only a part of fucoxanthin consumed DPPH and fucoxanthin was relatively less sensitive than β-carotene. Under a high oxygen pressure, e.g., 100 kPa, fucoxanthin had much less antioxidant activity than α-tocopherol for the oxidation of methyl linoleate in heptanol in the presence of 2,2′-azo-bis-iso-butyronitrile at 60 °C [59].

Sangeetha *et al*. [44,60] compared the effects of fucoxanthin and β-carotene on oxidative stress indicators (catalase, glutathione transferase, and Na^+^K^+^-ATPase) and the possible role in suppressing lipid peroxidation resulting from retinol deficiency in rats. The results showed that fucoxanthin and β-carotene protected cell membrane by decreasing Na^+^K^+^-ATPase activity and increasing the activities of catalase and glutathione transferase at the tissue and microsomal level affected by retinol deficiency possibly due to its antioxidant activity, thus protecting membranes against lipid peroxidation caused by retinol deficiency. The results also showed that fucoxanthin had greater potential than β-carotene and was somewhat more effective than retinol in reducing lipid peroxidation in plasma and liver resulting from retinol deficiency. Li *et al*. [61] showed that vitamin D_2_ was oxidized by singlet oxygen in the presence of riboflavin, light, and oxygen in a model system, and fucoxanthin and β-carotene could minimize the oxidation of vitamin D_2_ by quenching singlet oxygen.

The major structural differences in fucoxanthin and other carotenoids are the presence of an unusual allenic carbon (C-7′), 5,6-monoepoxide, two hydroxyl groups, a carbonyl group, and an acetyl group in the terminal ring of fucoxanthin [2,14]. The allenic bond was responsible for the higher antioxidant activity of fucoxanthin [27]. In addition, fucoxanthin has six oxygen atoms, and thus might be more sensitive to radicals, especially under anoxic conditions. It has been suggested that the antioxidant activity of carotenoids is correlated with the presence of intramolecular oxygen atoms [2].

It is worth mentioning that fucoxanthin also contains an α,β-unsaturated carbonyl group, and may function as a Michael acceptor which can react with important proteins such as Keap 1 in the Nrf2 system [62]. Liu *et al*. [62] recently showed that fucoxanthin enhanced HO-1 and NQO1 expression in murine hepatic BNL CL.2 cells through activation of the Nrf2/ARE system, and suggested that fucoxanthin might exert its antioxidant activity, at least partly, through its pro-oxidant actions.

### 3.2. Anti-Inflammatory Effects

Inflammatory response, a self-defensive reaction against various pathogenic stimuli, is characterized by attracting large amounts of leukocytes (neutrophiles, monocytes-macrophages, and mast cells) to the inflamed area, in which these inflammatory cells are triggered by inflammation mediators and generate superoxide anion and nitric oxide radicals, and may become a harmful self-damaging process [12,63]. Anti-inflammatory agents should reduce the inflammatory response by suppressing the productions of inflammatory cytokines such as tumor necrosis factor-α, interleukin-1β, and interleukin-6, and inflammatory mediators, including nitric oxide and prostaglandin E2 synthesized by inducible nitric oxide synthase and cyclooxygenase [64].

Heo *et al*. [22] and Kim *et al*. [65] authenticated the inhibitory effects of fucoxanthin on inflammatory cytokines and mediators in lipopolysaccharide-stimulated RAW 264.7 macrophages. These results showed that fucoxanthin inhibited the inducible nitric oxide synthase and cyclooxygenase 2 protein expressions, and reduced the levels of nitric oxide, prostaglandin E2, tumor necrosis factor-α, interleukin-1β, and interleukin-6 through the inhibition of nuclear factor-κB activation and the phosphorylation of mitogen-activated protein kinases [22,65].

Sakai *et al*. [66] indicated that fucoxanthin suppressed the degranulation of mast cells by inhibiting antigen-induced aggregation of high affinity IgE receptor followed by activation of the degranulating signals of mast cells, which played important roles in inflammation and immediate-type allergic reactions. Recently, Sakai *et al*. [67] evaluated the effect of carotenoids on dinitrofluorobenzene-induced contact hypersensitivity in mice to elucidate their effect on mast cell degranulation *in vivo*, and showed that fucoxanthin significantly inhibited ear swelling and reduced the levels of tumor necrosis factor-α and histamine, suggesting that fucoxanthin exerted an anti-inflammatory effect by suppressing mast cell degranulation *in vivo*.

Khan *et al*. [29,68] investigated the anti-inflammatory activity of methanol extract of *U. pinnatifida* against mouse ear edema and erythema induced by phorbol myristate acetate. The results showed that the extract suppressed edema and erythema with relative inhibition of 85 and 78%, respectively [68], and inhibited erythema by 50% when applied within 1 h before or 15 min after application of phorbol myristate acetate [29]. In addition, the extract suppressed the acetic acid-induced writhing response in the analgesic test, and also demonstrated antipyretic activity in yeast-induced hyperthermic mice [69].

### 3.3. Anticancer Activity

Apoptosis induction has been suggested to be the biochemical mechanism by which fucoxanthin exerted an inhibitory effect on tumor cells [23,25,31,70,71]. The apoptosis-inducing effect of fucoxanthin on human promyelocytic leukemia HL-60 cell line has been investigated by Hosokawa *et al*. [23], who found that fucoxanthin exhibited strong antiproliferative activity and could induce apoptosis of HL-60 cells. In HL-60 cells, fucoxanthin caused cleavages of procaspase-3 and poly-ADP-ribose polymerase, and apoptosis induction by fucoxanthin was mediated through mitochondrial membrane permeabilization and caspase-9 and caspase-3 activation [72]. Kim *et al*. [15] showed that fucoxanthin induced reactive oxygen species generation, inactivated the Bcl-xL signaling pathway, induced caspase-3, -7, and poly-ADP-ribose polymerase cleavage, and thus triggered the apoptosis of HL-60 cells, indicating that the generation of reactive oxygen species was a critical target in fucoxanthin-induced apoptosis in HL-60 cells.

It had been reported that β-carotene did not show an apoptosis-inducing effect on HL-60 cells, indicating that the carotenoid structure might be crucial for inducing apoptosis [23]. Recently, Ganesan *et al*. [73] showed that fucoxanthin, astaxanthin, siphonaxanthin, neoxanthin, and violaxanthin had significant cytotoxic effects against cultured HL-60 cells and the inhibitory effect of fucoxanthin on the viability of HL-60 cells was remarkable compared with astaxanthin, β-carotene, zeaxanthin, lutein, *etc*. Both halocynthiaxanthin and fucoxanthinol showed remarkable induction of apoptosis in HL-60 cells, MCF-7 breast cancer cells, and Caco-2 colon cancer cells, and the antiproliferative and apoptosis-inducing effects of halocynthiaxanthin and fucoxanthinol on these cells were significantly greater than those of fucoxanthin, possibly at least partly, related to the hydroxyl group in halocynthiaxanthin and fucoxanthinol [49]. Both fucoxanthinol and amarouciaxanthin A also reduced the viability of PC-3 human prostate cancer cells, and the 50% inhibitory concentrations of fucoxanthin, fucoxanthinol, and amarouciaxanthin A on the proliferation of PC-3 cells were 3.0, 2.0, and 4.6 μM, respectively, indicating that the 5,6-epoxide in fucoxanthin and fucoxanthinol played important roles in cytotoxicity, and other mechanisms might be also involved in the antiproliferative effect of epoxycarotenoids on PC-3 cells [42].

Adult T-cell leukemia is an incurable malignancy of mature CD4^+^ T cells caused by human T-cell leukemia virus type 1. Ishikawa *et al*. [53] assayed the antiproliferative effects of some carotenoids such as fucoxanthin, fucoxanthinol, β-carotene and astaxanthin, and found that both fucoxanthin and fucoxanthinol had remarkable antiproliferative effects on human T-cell leukemia virus type 1-infected T-cell lines and adult T-cell leukemia cells in vitro, and β-carotene and astaxanthin had mild inhibitory effects.

The exposure of human non-small-cell bronchopulmonary carcinoma line NSCLC-N6 and human lung epithelial cell line A549 to fucoxanthin distinctly induced morphological change such as rounding up, reduction of cell volume, chromatin condensation, nuclei fragmentation, and formation of apoptotic bodies for the two bronchopulmonary cells lines, and suggested that fucoxanthin could trigger the terminal differentiation of cancerous cells *in vitro* [36].

It was reported that fucoxanthin reduced the viability of human colon cancer cell lines Caco-2, HT-29, and DLD-1 cells (Caco-2 > DLD-1 > HT-29) and induced apoptosis in a dose- and time-dependent manner, while astaxanthin and β-carotene were not found to change the viability of Caco-2 cells. The decreased expression level of Bcl-2 protein might contribute to fucoxanthin-induced apoptosis in Caco-2 cells [25]. Kotake-Nara *et al*. [71] evaluated the effects of fucoxanthin and neoxanthin on the viability of human colorectal carcinoma Caco-2, human colorectal adenocarcinoma HCT116 cell lines, mouse melanoma B16, human normal embryonic lung fibroblast MRC-5, and human male umbilical cord fibroblast HUC-Fm cell lines. The results showed that fucoxanthin and neoxanthin had nearly the same actions on the cultured cells and were more effective in reducing the viability of HCT116 cancer cells than that of the other cancer and normal cell lines, while lycopene was found to be less effective. Das *et al*. [74] indicated that fucoxanthin inhibited the proliferation of human colon cancer cell lines WiDr and HCT116 cells by inducing cell cycle arrest at the G0/G1 phase through up-regulating the cyclin-dependent kinase inhibitory protein p21^WAF1/Cip1^ and retinoblastoma protein (pRb).

Kotake-Nara *et al*. [70] showed that fucoxanthin significantly reduced the viability of three human prostate cancer cell lines PC-3, DU 145 and LNCaP to 14.9%, 5.0%, and 9.8%, respectively, through apoptosis induction in these cancer cells. The succeeding study demonstrated that fucoxanthin decreased the levels of Bax and Bcl-2 proteins, and induced apoptosis in PC-3 cells through caspase-3 activation [71,75]. Satomi and Nishino [76] showed that fucoxanthin induced cell cycle arrest at the G1 phase and GADD45A expression, and inhibited the growth of DU145 cells. The results suggested that GADD45A and Pim-1 protooncogene might be important genes associated with the action of fucoxanthin, and GADD45A might be involved in fucoxanthin-induced G1 arrest. In the subsequent study, Satomi and Nishino [77] showed that several MAPKs modulated the induction of GADD45 and G1 arrest, and positive regulation by SAPK/JNK was involved in GADD45A induction and G1 arrest by fucoxanthin, indicating that GADD45A was closely related with the G1 arrest induced by fucoxanthin, and MAPK pathways were implicated in fucoxanthin-induced GADD45A expression and G1 cell cycle arrest in tumor cells depending on the cell type.

Satomi and Nishino [76] previously showed that fucoxanthin inhibited the growth of human hepatocellular carcinoma HepG2 cells. In the subsequent study, Satomi and Nishino [77] investigated the role of MAPKs in the induction of G1 arrest and GADD45 expression by fucoxanthin in HepG2 cells, and showed that the inhibitions of p38 MAPK and ERK1/2 MAPK enhanced the induction of GADD45A and GADD45B expressions, respectively, and GADD45A was an important factor in the induction of G1 arrest by fucoxanthin in HepG2 cells. The molecular mechanisms of fucoxanthin against hepatocellular carcinoma using HepG2 cells had been studied by Das *et al*. [16], who found that the growth-inhibitory effect of fucoxanthin on the cancer cells was chiefly due to an arrest in the G0/G1 phase of the cell cycle, and no apoptosis was observed, indicating that fucoxanthin possessed cytostatic rather than cytocidal activity in HepG2 cells. This study suggested that both the proteolysis and transcriptional suppression might be responsible for the decreasing levels of cyclin Ds and the suppression of cyclin D/cdk4 activity in fucoxanthin-treated HepG2 cells and might be related to the antitumor activity. Liu *et al*. [30] indicated that fucoxanthin effectively inhibited the proliferation of human hepatoma SK-Hep-1 cells but facilitated the growth of murine embryonic hepatic BNL CL.2 cells. The study found that fucoxanthin significantly increased protein and mRNA expressions of connexin 43 and connexin 32 in SK-Hep-1 cells, and thus enhanced gap junctional intercellular communication of SK-Hep-1 cells, which might be responsible for the increase of the intracellular calcium level, leading to cell cycle arrest at G0/G1 phase, DNA fragmentation, and apoptosis of SK-Hep-1 cells.

Recently, the effects of fucoxanthin on human gastric adenocarcinoma MGC-803 cells were investigated by Yu *et al*. [78], who found that fucoxanthin induced cell cycle arrest in G2/M phase and apoptosis of MGC-803 cells, and down-regulated the expressions of CyclinB1 and survivin in MGC-803 cells. The study also showed that fucoxanthin might reduce CyclinB1 expression through JAK/STAT signal pathway, and thus inhibit proliferation of MGC-803 cells.

Bladder cancer is not only a serious malignancy but also the most expensive cancer to survey and treat. Zhang *et al*. [17] showed that fucoxanthin exhibited remarkable antiproliferative effects on human urinary bladder cancer EJ-1 cells and reduced the viability of EJ-1 cells by inducing apoptosis, which was characterized by morphological changes, DNA ladder, and increased percentage of hypodiploid cells, and activating caspase-3 activity with a maximum ratio of apoptotic cells of >93% with 20 μM fucoxanthin.

Primary effusion lymphoma is a very aggressive type of non-Hodgkin’s lymphoma infected by human herpesvirus 8. It was found that fucoxanthin and fucoxanthinol decreased cell viability in primary effusion lymphoma BCBL-1 and TY-1 cells. Fucoxanthin and fucoxanthinol induced cell cycle arrest during G1 phase and caspase-dependent apoptosis, inhibited the activation of nuclear factor-κB, activator protein-1, and phosphatidylinositol 3-kinase/Akt pathways, and down-regulated anti-apoptotic proteins and cell cycle regulators in primary effusion lymphoma cells. In addition, fucoxanthin reduced the growth of primary effusion lymphoma cells in the xenografted mice, suggesting that fucoxanthin could be potentially effective for the treatment of primary effusion lymphoma [54].

An earlier study indicated that fucoxanthin inhibitd the growth of the human neuroblastoma GOTO cells by causing the arrest in the G0–G1 phase of cell cycle and decreasing N-*myc* gene expression [79]. Subsequently, Nishino *et al*. [48] found that halocynthiaxanthin showed a more potent inhibitory effect on the growth of human neuroblastoma GOTO cells, and also inhibited the growth of other human malignant tumor cells.

The antiproliferative effect of fucoxanthin is dependent on its isomeric structure. Nakazawa *et al*. [31] found that the antiproliferative activity of 13-*cis* and 13′-*cis* fucoxanthin was significantly higher than that of the all-*trans* or 9′-*cis* isomeric forms on the growth of cancer cells. In addition, 13′-*cis* fucoxanthin had greatest inhibitory effect on the growth of HL-60 cells, followed by 13-*cis* isomer and all-*trans* or 9′-*cis* isomers. It was suggested that the stronger antiproliferative and inhibitory effect of *cis*-fucoxanthin might be due to the steric hindrances offered by their structure.

In animal experiment, fucoxanthin significantly inhibited the formation and development of aberrant crypt foci, a preneoplastic marker for colon cancer, induced by azoxymethane [80] and 1,2-dimethylhydrazine dihydrochloride [81] in mice. Fucoxanthin had been proven to suppress spontaneous liver tumorigenesis in C3H/He male mice and showed antitumor-promoting activity in a two-stage carcinogenesis experiment in the skin of ICR mice, initiated with 7,12-dimethylbenz[*a*]anthracene and promoted with 12-*O*-teradecanoylphorbol-13-acetate and mezerein [13]. In addition, fucoxanthin was reported to inhibit duodenal carcinogenesis induced by *N*-ethyl-*N*′-nitro-*N*-nitrosoguanidine in mice [82].

Although the antitumor effects of fucoxanthin are known, the precise mechanism of action has yet to be elucidated [76]. The anticancer activity of fucoxanthin was partly based on the regulative effect of fucoxanthin on biomolecules related to cell cycle and apoptosis [83]. In addition, fucoxanthin was found to be able to selectively inhibit the mammalian DNA polymerase activities, especially replicative DNA polymerases (*i.e.*, pol α, δ, and ɛ), and thus had anti-neoplastic activity [20]. Further investigations are needed to assess the details of the molecular mechanisms of fucoxanthin against different types of cancer cells with animal models [83].

### 3.4. Anti-Obese Effect

An excessive accumulation of fat in the body and white adipose tissue causes obesity and a disturbance of cytokine secretion from adipose tissue, and thus results in an increased risk of many serious diseases such as type II diabetes, hyperlipidemia, hypertension, and cardiovascular disease [26,50,84].

Maeda *et al*. [26] firstly reported that lipids from *U. pinnatifida* reduced abdominal white adipose tissue weights in Wistar rats and KK-Ay mice. Miyata *et al*. [18] showed that *L. japonica* and *L. ochotensis* inhibited fat absorption, decreased the serum triglyceride level in SD rats, and had an anti-obesity effect on ddY mice induced by a high-fat diet. It was suggested that fucoxanthin was an active component for the antiobesity effect [26]. Many studies have shown that fucoxanthin, even with a 0.02% dose [85], significantly lowered body weight [43,86–88], body fat accumulation [89], visceral fat-pads weights [85,87], white adipose tissue weight gain [50,88–90], and the size of adipocyte [85] in diabetic/obese KK-Ay mice, high-fat diet-induced obese C57BL/6N mice or C57BL/6J mice, and increased brown adipose tissue weight in KK-Ay mice [50]. However, fucoxanthin did not affect white adipose tissue weight gain in lean C57BL/6J mice [90]. It was found that fucoxanthin significantly reduced plasma and hepatic triglyceride concentrations and the activities of adipocytic fatty acid synthesis, hepatic fatty acid and triglyceride synthesis, and cholesterol-regulating enzymes, and significantly increased the concentrations of plasma high-density lipoprotein-cholesterol, fecal triglyceride and cholesterol, as well as fatty acid oxidation enzyme activity in epididymal white adipose tissue of mice, indicating that fucoxanthin ameliorated the plasma and hepatic lipid profile, fecal lipids and body fat mass, hepatic cholesterol metabolism, fatty acid synthesis, and lipid absorption [57,88]. Moreover, fucoxanthin significantly lowered mRNA expression of proliferators activated receptor γ and the activity of hepatic phosphatidate phosphohydrolase, and significantly increased the mRNA expressions of β-oxidation-related acyl-coA oxidase 1, palmitoyl and proliferators activated receptor α [57]. In addition, fucoxanthin and fucoxanthinol inhibited both lymphatic triglyceride absorption and the increase of triglyceride concentration in systemic blood, likely due to their inhibitory effects on lipase activity in the gastrointestinal lumen [91].

The potential anti-obesity effect might be mediated by improving plasma adipokine level (e.g., lowered leptin level and elevated adiponectin level), down-regulating various lipogenic enzyme activities and fat production, up-regulating fatty acid β-oxidation activity and uncoupling protein gene expressions in visceral adipose tissues, suggesting that fucoxanthin might act as a regulator of lipid metabolism in fat tissues [87]. Mitochondrial uncoupling protein 1, usually expressed only in brown adipose tissue that occurs little in adult humans, is a key molecule for metabolic thermogenesis to avoid an excess of fat accumulation [26]. Many studies suggested that the antiobesity effect of fucoxanthin was due to oxidation of fatty acids, heat production, and energy dissipation through upregulating the expression of uncoupling protein 1 in the white adipose tissue [26,50,51,86,88,92], and thus inducing the decrease in abdominal fat in mice [86]. Recently, Miyashita *et al*. [83] reported that fucoxanthin promoted mRNA expression of β_3_-adrenergic receptor in white adipose tissue of obese mice, which was responsible for lipolysis and thermogenesis. The regulatory effect of fucoxanthin on peroxisome proliferator-activated receptor γ expression in adipose tissue was an important modulator for uncoupling protein 1 expression. Leptin is mainly produced in adipocyte and controls body weight and fat weight by regulating the food intake and energy expenditure. Fucoxanthin remarkably reduced leptin mRNA in white adipose tissue and the level of plasma leptin in KK-Ay mice [50] and high fat diet-induced obese C57BL/6J mice [85,89]. The lower leptin level might reflect the decrease in the size of white adipose tissue induced by fucoxanthin [50]. In addition, Sugawara *et al*. [93] found that fucoxanthin had significant antiangiogenic activity, which was also responsible for its anti-obesity effect.

Maeda *et al*. [84] showed that fucoxanthin and fucoxanthinol inhibited intercellular lipid accumulation and decreased glycerol-3-phosphate dehydrogenase activity during adipocyte differentiation of 3T3-L1 cells. The suppressive effect of fucoxanthinol on the differentiation of murine 3T3-L1 preadipocytes to adipocytes was stronger than that of fucoxanthin [84], and the suppressive effect of amarouciaxanthin A, a dominant metabolite of fucoxanthin in white adipose tissue, was stronger than that of fucoxanthinol [94]. Amarouciaxanthin A markedly down-regulated the mRNA expressions of adipocyte fatty acid-binding protein, lipoprotein lipase, and glucose-transporter 4 in 3T3-L1 cells [94]. Kang *et al*. [95] found that that fucoxanthin inhibited 3T3-L1 adipocyte differentiation at intermediate (days 2–4) and late stages (days 4–7), whereas it enhanced 3T3-L1 adipocyte differentiation at an early stage (days 0–2) by modulating the expression of key adipogenic transcriptional regulators such as peroxisome proliferator-activated receptor γ, CCAAT/enhancer-binding protein α, and sterol regulatory element-binding protein 1c, and inhibited glucose uptake by suppressing the phosphorylation of insulin receptor substrate 1 in mature 3T3-L1 adipocytes, suggesting that fucoxanthin exerted anti-obesity effect by inhibiting the expression of key transcriptional regulators at intermediate and late stages and glucose uptake in mature adipocytes. Noticeably, some non-allenic carotenoids, such as lutein, lutein epoxide, α-carotene, and β-carotene 5,6-epoxide, were found to not exhibit the suppressive effect on adipocyte differentiation in 3T3-L1 cells, indicating that the allene bond of fucoxanthin and its metabolites might be important for the inhibition [83,92,94,96].

Maeda *et al*. showed that, although no significant uncoupling protein 1 expression in the white adipose tissue of KK-Ay mice fed fish oil [50] or medium-chain triacylglycerols [51] was found, the anti-obesity effect of fucoxanthin was increased by dissolving fucoxanthin in fish oil or medium-chain triacylglycerols, indicating the combination of fucoxanthin and lipids is more effective for attenuating the weight gain of the white adipose tissue than feeding with fucoxanthin alone, and suggesting that the presence of lipids can increase the absorption rate of fucoxanthin in KK-Ay mice [50,51]. Recently, Okada *et al*. [32] developed a lipid delivery system, and suggested that incorporation of *U. pinnatifida* lipid into phospholipid by means of capsulation might lead to an additive increase in the antiobesity effect of fucoxanthin on KK-Ay mice.

In a 16-week clinical trial, Abidov *et al*. [52] investigated the effects of orally administered the mixture (Xanthigen) of fucoxanthin and pomegranate seed oil on body weight, body fat, liver lipids, blood biochemistry, and resting energy expenditure in obese, non-diabetic premenopausal women diagnosed with non-alcoholic fatty liver disease or presenting with normal liver fat. The results showed that Xanthigen reduced body weight (from 94.1 ± 2.1 and 94.5 ± 2.1 to 87.2 ± 3.7 and 88.2 ± 1.9 kg for volunteers with fatty liver and normal liver, respectively), body fat (from 42.3 ± 2.2 and 43.3 ± 2.9 to 37.9 ± 2.9 and 38.1 ± 3.2 kg, respectively), and liver fat content (from 15.3 ± 4.1 and 5.1 ± 1.5 to 9.4 ± 3.1 and 3.4 ± 1.8%, respectively), improved liver function tests in obese non-diabetic women, and increased resting energy expenditure.

### 3.5. Antidiabetic Activity

Maeda *et al*. [50] found that fucoxanthin markedly decreased the blood glucose and plasma insulin levels, as well as water intake in diabetic/obese KK-Ay mice. It was suggested that fucoxanthin improved insulin resistance and decreased blood glucose level, at least in part, through downregulating adipokines such as tumor necrosis factor-α, monocyte chemoattractant protein-1, interleukin-6, and plasminogen activator inhibitor-1 via downregulating their mRNA expression by directly acting on adipocytes and macrophages in white adipose tissue and up-regulation of glucose transporter 4 in skeletal muscle in KK-Ay mice [50,83,89,90,92].

Hosokawa *et al*. [90] demonstrated that fucoxanthin attenuated hyperglycemia in KK-Ay mice, but did not affect blood glucose levels in lean C57BL/6J mice. However, a high-fat feeding could prompt obesity, hyperinsulinemia, high blood glucose, insulin resistance, and non-alcoholic fatty liver disease in C57BL/6J mice [85]. Maeda *et al*. [89] and Park *et al*. [85] showed that fucoxanthin significantly lowered the fasting blood glucose concentration, the plasma insulin level, and the insulin resistance index in diet-induced obese mice. Fucoxanthin might improve altera–tions in lipid metabolism and insulin resistance induced by a high fat diet, at least in part, through reducing visceral fat mass, hyperinsulinemia, hepatic glucose production, and hepatic lipogenesis, and altering hepatic glucose-regulating enzymes activities [85]. Moreover, Woo *et al*. [57] demonstrated that fucoxanthin significantly reduced the blood glucose, hemoglobin A_1c_, plasma insulin, and resistin levels, and no change was found in the plasma glucagon concentration in high-fat diet fed C57BL/6N mice, indicating that the reduction in the insulin/glucagon ratio could be in part responsible to lowering blood glucose concentration by fucoxanthin.

### 3.6. Hepatoprotective Effect

Woo *et al*. [57] found that fucoxanthin significantly lowered the hepatic lipid contents, while feces weight and fecal lipids significantly increased by inhibiting lipid adsorption in high-fat diet fed C57BL/6N mice. Park *et al*. [85] also demonstrated that fucoxanthin significantly decreased the hepatic lipid droplet accumulation in high-fat fed mice, possibly through reducing the activity of hepatic fatty acid synthesis-related enzymes [57,85]. Furthermore, it was shown that there were no significant dose-dependent effects on hepatic lipid changes by 0.05% and 0.2% fucoxanthin and the 0.05% fucoxanthin might be sufficient for improving the hepatic lipid content [57], while no significant change was found in the plasma lipids in Wister rats [86]. In addition, fucoxanthin significantly up-regulated glycolytic enzyme such as glucokinase in the liver, and thus increased the ratio of hepatic glucokinase/glucose-6-phosphatase and glycogen content, indicating that fucoxanthin normalized the hepatic glycogen content in high-fat fed mice [85].

The reduction of liver lipids might be due to the increase of docosahexaenoic acid, which reduces the activity of hepatic enzymes in fatty acid synthesis and increases hepatic fatty acid β-oxidation, in the liver [86]. Tsukui *et al*. [97] first reported that fucoxanthin and fucoxanthinol enhanced the amount of docosahexaenoic acid in the liver of KK-Ay mice, whereas the level of docosahexaenoic acid in the small intestine remained unaltered. Subsequently, Tsukui *et al*. [43] confirmed that fucoxanthin increased the amount of docosahexaenoic acid in the liver of normal adult C57BL/6J mice. In addition, an increase in arachidonic acid (ω-6) was also found in fucoxanthin-fed mice, indicating that fucoxanthin might modify the metabolic pathways of ω-3 and ω-6 highly unsaturated fatty acids. Recently, Airanthi *et al*. [10] showed that the levels of docosahexaenoic acid and arachidonic acid in liver lipids of KK-Ay mice given the lipids from brown seaweeds significantly increased.

Furthermore, Liu *et al*. [98] investigated the effects of fucoxanthin on hepatocyte lipid peroxidation and hepatotoxicity induced by ferric nitrilotriacetate in murine embryonic hepatic BNL CL.2 cells, and showed that fucoxanthin significantly recovered cell proliferation and increased the levels of glutathione. Moreover, fucoxanthin significantly decreased intracellular reactive oxygen species and DNA damage, and markedly decreased the level of thiobarbituric acid-reactive substances and protein carbonyl contents in BNL CL.2 cells induced by ferric nitrilotriacetate, indicating that fucoxanthin effectively protected against ferric nitrilotriacetate-induced hepatotoxicity by decreasing intracellular reactive oxygen species, thiobarbituric acid-reactive substances, and protein carbonyl contents, and increasing glutathione level, associated with the antioxidant effects of fucoxanthin.

### 3.7. Skin-Protective Effect

Over exposure to ultraviolet radiation from sunlight leading to the generation of reactive oxygen species, inflammatory reaction, and angiogenesis of the skin is presumed to be the primary causative agent in the damage of cellular constituents and some cutaneous disease such as pigmentation, laxity, wrinkling, erythema, and skin carcer [99,100]. Heo and Jeon [99] revealed that fucoxanthin significantly decreased intracellular reactive oxygen species generated by exposure to ultraviolet B radiation in human fibroblast. Fucoxanthin elevated cell survival rate and inhibited cell damage for pre-treated cells, indicating that fucoxanthin could protect skin against photodamage induced by ultraviolet B irradiation from sunlight. Shimoda *et al*. [101] found that fucoxanthin inhibited tyrosinase activity, melanogenesis in melanoma, and ultraviolet B-induced skin pigmentation. The results showed that fucoxanthin significantly suppressed expression of cyclooxygenase-2, endothelin receptor A, p75 neurotrophin receptor, prostaglandin E receptor 1, melanocortin 1 receptor and tyrosinase-related protein 1, suggesting that fucoxanthin presented anti-pigmentary activity by topical or oral application in ultraviolet B-induced melanogenesis possibly through the suppression of prostaglandin E_2_ synthesis and melanogenic stimulant receptors. Recently, Urikura *et al*. [100] showed that fucoxanthin significantly suppressed ultraviolet B-induced epidermal hypertrophy, which may cause wrinkle formation, vascular endothelial growth factor, matrix metalloproteinases-13 expression, and the increase of thiobarbituric acid reactive substances in the skin of hairless mice. The results indicated that topical treatment with fucoxanthin prevented skin photoage and wrinkle formation in ultraviolet B-irradiated hairless mice, possibly through the antioxidant and antiangiogenic effects of fucoxanthin. These studies suggest that fucoxanthin may be an effective ultraviolet protcect ingredient able to be used in cosmetic and sunscreen in protecting skin from photoaging. Moreover, it would be worthy to probe into the effect of oral administration of fucoxanthin on skin photoage.

### 3.8. Antiangiogenic Effect

Sugawara *et al*. [93] investigated the antiangiogenic effects of fucoxanthin using cultured human umbilical vein endothelial cells and the rat aortic ring, and found that fucoxanthin could significantly suppress the differentiation of endothelial progenitor cells into endothelial cells and the formation of new blood vessels, and significantly reduced the tube length of endothelial cells. The results showed that fucoxanthin and fucoxanthinol inhibited microvessel outgrowth in an ex vivo angiogenesis assay using a rat aortic ring, suggesting that the antiangiogenic effect of fucoxanthin might be useful in preventing angiogenesis-related diseases, such as cancer, diabetic retinopathy, atherosclerosis and psoriasis.

### 3.9. Cerebrovascular Protective Effect

Ikeda *et al*. [24] revealed that the brown seaweed *U. pinnatifida* significantly delayed the development of stroke signs with no change in blood pressure and increased the life span of salt-loaded hypertensive rats. In addition, the preventive effect of fucoxanthin on cultured neuronal cells from hypertensive rats was also investigated by Ikeda *et al*. [24], who found that fucoxanthin markedly attenuated neuronal cell injury in hypoxia and re-oxygenation, possibly through its radical-scavenging activity, and suggested that fucoxanthin had a beneficial effect on cerebrovascular diseases against ischaemic neuronal cell death in stroke-prone spontaneously hypertensive rats.

### 3.10. Bone-Protective Effect

Using cells from the macrophage cell line RAW264.7 able to differentiate into osteoclast-like cells when stimulated by receptor activator of nuclear factor κB ligand, Das *et al*. [41] investigated the effects of fucoxanthin on the osteoclast differentiation of precursor RAW264.7 monocytes and its cytotoxicity against the osteoclast-like cells differentiated from RAW264.7 cells and the osteoblast-like cell line MC3T3-E1 cells. The results showed that fucoxanthin significantly suppressed the differentiation of RAW264.7 cells with no toxicity to RAW264.7 cells, induced apoptosis through the activation of caspase-3 and sequentially the cleavage of poly-ADP-ribose polymerase in osteoclast-like cells, and did not decrease cell viability in the osteoblast-like cell line MC3T3-E1, indicating that the cytotoxicity of fucoxanthin against osteoclasts was stronger than that against osteoblasts. The study suggested that fucoxanthin suppressed osteoclastogenesis through inhibiting osteoclast differentiation and inducing apoptosis in osteoclasts, but did not antagonize bone formation, and fucoxanthinol might play an important role in inducing apoptosis in osteoclast-like cells and exert a suppressive effect on osteoclastogenesis. These results indicate that fucoxanthin is helpful for the prevention of bone diseases such as osteoporosis and rheumatoid arthritis.

### 3.11. Ocular Protective Effect

After-cataract, also known as posterior capsule opacification, is the main long term complication of extracapsular cataract extraction due to the proliferation and migration of lens epithelial cells left in the capsular bag after cataract surgery [102]. The growth of human lens epithelial cell line SRA 01/04 was apparently inhibited by fucoxanthin, indicating that fucoxanthin was an efficient and safe antiproliferative agent for human lens epithelial cell line and might be applied to the formulation of ocular implant products used in cataract treatment for the prevention of after-cataract [36].

In addition, Shiratori *et al*. [103] studied the anti-ocular inflammatory effect of fucoxanthin on lipopolysaccharide-induced uveitis in male Lewis rats, and found that fucoxanthin suppressed the development of the uveitis.

### 3.12. Antimalarial Effect

Malaria, widespread in tropical and subtropical regions, is a mosquito-borne infectious disease of humans caused by *Plasmodium falciparum*, a one-celled apicomplexan parasite. Afolayan *et al*. [21] first found that organic extract from brown seaweed *S. heterophyllum* exhibited promising antiplasmodial activity, and thus separated sargaquinoic acid, sargahydroquinoic acid, sargaquinal, and fucoxanthin from the extract for further investigation. The results indicated that fucoxanthin showed the highest antiplasmodial activity, while sargaquinal showed good antiplasmodial activity, and sargaquinoic acid and sargahydroquinoic acid were only moderately active. Although the antiplasmodial activities of four compounds might be related to their antioxidant properties, further studies were required in order to clarify their mode of action.

## 4. Conclusions

Growing evidence from tissue culture and animal experiment suggests that fucoxanthin has potential health promoting effects. Fucoxanthin has attracted considerable interest because of its potent bioactivities, including its antioxidant, anti-inflammatory, anticancer, anti-obese, antidiabetic, antiangiogenic, and antimalarial activities, and its protective effects on the liver, blood vessels of the brain, bones, skin, and eyes. Particularly, the anti-obese effect, antiproliferative effects on adult T-cell leukemia cells [53], and inhibitory effect on the viability of HL-60 cells [73] of fucoxanthin are distinctly more potent than that of β-carotene and astaxanthin. The unique suppressive effect of fucoxanthin on adipocyte differentiation is related to its structural properties, where an allenic bond is essential for the expression of this activity, and carotenoids without an allenic bond are not active [83].

Orally-administered fucoxanthin is metabolized into fucoxanthinol and amarouciaxanthin A in mice. Therefore, fucoxanthinol, amarouciaxanthin A, or other metabolites of fucoxanthin in human should be considered in mechanistic studies of the biological actions of fucoxanthin. Although some brown seaweeds containing high levels of fucoxanthin are the most common edible delicacies, some studies showed that the bioavailability of fucoxanthin in brown seaweeds was low in humans. However, dietary combination of fucoxanthin isolated from brown seaweeds or diatoms and edible oil or lipid could increase the absorption rate of fucoxanthin, and might be developed as a promising marine drug. More extensive animal experimentation and well-controlled clinical trials are suggested for further studies.
